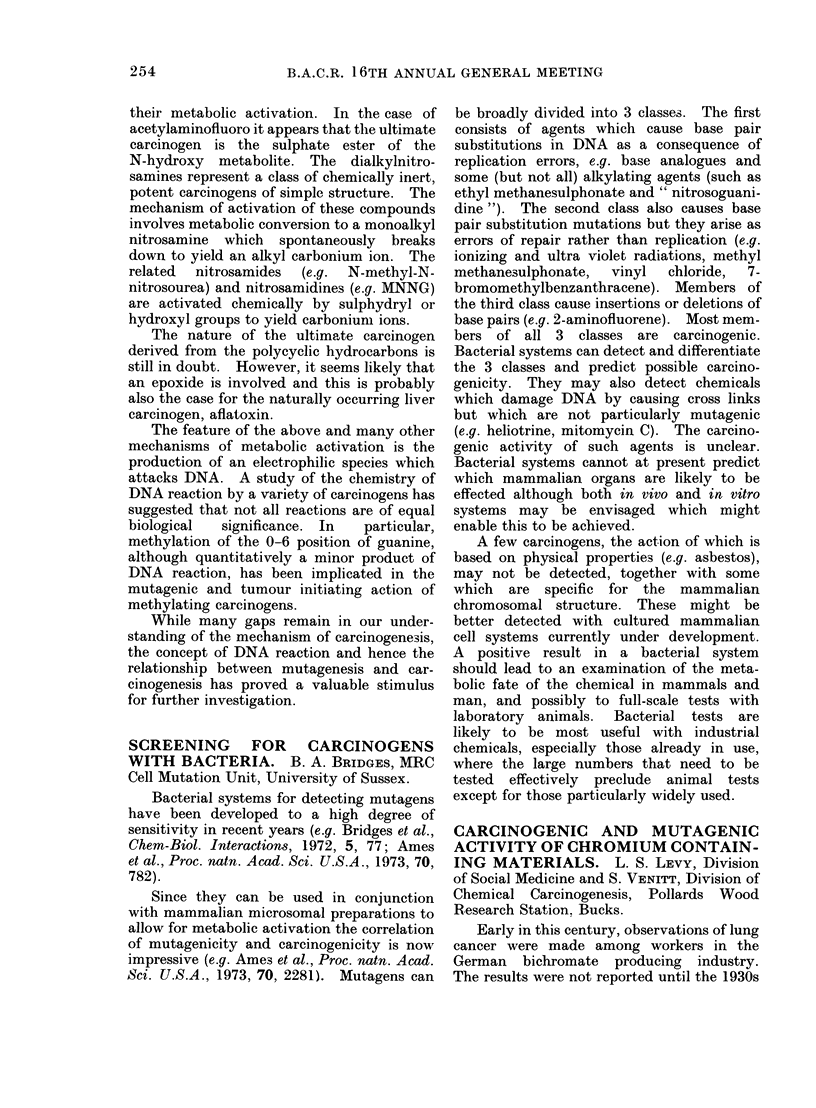# Proceedings: Screening for carcinogens with bacteria.

**DOI:** 10.1038/bjc.1975.200

**Published:** 1975-08

**Authors:** B. A. Bridges


### SCREENING FOR CARCINOGENS WITH BACTERIA.

B. A. Bridges, MRC Cell Mutation Unit, University of Sussex.

Bacterial systems for detecting mutagens have been developed to a high degree of sensitivity in recent years (*e.g.* Bridges *et al.*, *Chem-Biol. Interactions*, 1972, **5**, 77; Ames *et al.*, *Proc. natn. Acad. Sci. U.S.A.*, 1973, **70**, 782).

Since they can be used in conjunction with mammalian microsomal preparations to allow for metabolic activation the correlation of mutagenicity and carcinogenicity is now impressive (*e.g.* Ames *et al.*, *Proc. natn. Acad. Sci. U.S.A.*, 1973, **70**, 2281). Mutagens can be broadly divided into 3 classes. The first consists of agents which cause base pair substitutions in DNA as a consequence of replication errors, *e.g.* base analogues and some (but not all) alkylating agents (such as ethyl methanesulphonate and " nitrosoguanidine "). The second class also causes base pair substitution mutations but they arise as errors of repair rather than replication (*e.g.* ionizing and ultra violet radiations, methyl methanesulphonate, vinyl chloride, 7-bromomethylbenzanthracene). Members of the third class cause insertions or deletions of base pairs (*e.g.* 2-aminofluorene). Most members of all 3 classes are carcinogenic. Bacterial systems can detect and differentiate the 3 classes and predict possible carcinogenicity. They may also detect chemicals which damage DNA by causing cross links but which are not particularly mutagenic (*e.g.* heliotrine, mitomycin C). The carcinogenic activity of such agents is unclear. Bacterial systems cannot at present predict which mammalian organs are likely to be effected although both *in vivo* and *in vitro* systems may be envisaged which might enable this to be achieved.

A few carcinogens, the action of which is based on physical properties (*e.g.* asbestos), may not be detected, together with some which are specific for the mammalian chromosomal structure. These might be better detected with cultured mammalian cell systems currently under development. A positive result in a bacterial system should lead to an examination of the metabolic fate of the chemical in mammals and man, and possibly to full-scale tests with laboratory animals. Bacterial tests are likely to be most useful with industrial chemicals, especially those already in use, where the large numbers that need to be tested effectively preclude animal tests except for those particularly widely used.